# An Orthopedic-, Surgical-, and Epidemiological-Based Investigation of Leprosy, in the Tamil Nadu State of India

**DOI:** 10.1155/2012/783853

**Published:** 2012-05-17

**Authors:** Jason Samona, Scott Samona, Cameron Samona, S. Gopalakrishnan, P. Shekhar, D. Kubern, P. S. Mohan Kumar, Reza Nassiri

**Affiliations:** ^1^College of Osteopathic Medicine, Michigan State University, East Lansing, MI 48824, USA; ^2^College of Human Medicine, Michigan State University, East Lansing, MI 48824, USA; ^3^Oakland University, Rochester, MI 48309, USA; ^4^Department of Community Medicine, SRM Medical College, SRM University, Tamil Nadu, Kattankulathur 603 203, India; ^5^Michigan State University, Institute of International Health (MSU-IIH), B-319 West Fee Hall, East Lansing, MI 48824, USA

## Abstract

No other research paper has ever been written about leprosy in this manner. The orthopedic and surgical implications, as well as the functional debility caused by the disease, have not been previously explained by past research as they have in such a comprehensive manner in this paper. The results of this study have regional and global implications as they pertain to disease pathology, risk factor recognition/disease prevention, and treatment. This paper is a unique, in that it also serves as a combination of a review of the current medical literature, as well as an epidemiological survey of the disease in a region of the world which has never been researched in the past. Clinical data points to the possibility of a new strain of the disease. This information is of significance because it effects prevention and improved treatment of the disease, which leads to devastating sequela. This was a cross-sectional study involving subjects diagnosed with leprosy in the Chengalpet region of the Kancheepuram District, of the Tamil Nadu state of India. The study was performed at the Tamil Nadu Medical College Teaching Hospital and Research Center. This study included various physical examinations, observation and survey of lesions, questionnaires in regard the debilitating orthopedic and medical effects of the disease, as well as treatment options.

## 1. Background and Purpose

This paper is a unique, in that it is a combination of a review of the current medical literature, as well as an epidemiological review and survey of the disease in a region of the world which has never been researched in the past. The review of the literature presents information discovered in recently performed clinical studies, government and health care run epidemiological reviews, as well as historical facts about the disease. In order to gain a full understanding of the disease process, the investigation also reviewed epidemiological factors. India is home to the largest number of leprosy patients on earth. Although leprosy research has been conducted in India in the past, the Chengalpet region completely lacks any previous investigations. This paper also serves as a research paper revealing unique clinical information. No other paper has ever been written about leprosy and its orthopedic effects in this manner, ever before. This is a comprehensive study of the disease, especially focusing on the orthopedic considerations, risk factors, and treatments. The results of this study have regional and global implications as they pertain to disease pathology, risk factor recognition/disease prevention, and treatment. This disease rarely receives orthopedic attention, and it is a unique topic of discussion.

## 2. Methods

In this observational cross-sectional study, we have examined diagnosed leprosy patients at a medical college hospital and research center located in the Chengalpet region of Kancheepuram District, Tamil Nadu state of India. The hospital is located in a rural area and it is the only medical facility within a 16-mile radius. The facility serves a population of 15,000 individuals where over 100 individuals are confirmed to suffer from the disease of leprosy. The study was conducted on patients attending the outpatient department. All the subjects were of Indian descent and were born in India. Data was collected over a 60-day period at the hospital/research center, from approximately the months of January of 2011 until March of 2011. The subjects of this study were lifelong residents of the region and regular patients of this medical facility. They received all of their medical treatment from this hospital, as confirmed by questioning of the patient, the medical staff who were familiar with the patients, and review of medical records. Therefore we can claim that the vast majority of the treatment and disease progression was supervised at this one hospital. All leprosy patients who came to the hospital during the 60-day study were asked if they would be willing to participate in this study, therefore no specific selection process used. Therefore this avoids bias as to who was chosen as subjects in the study and also displays an accurate representation of the overall leprosy population in this region via random selection process.

Information was collected by 3 physicians working at the hospital, with a history of diagnosing and treating the disease of leprosy in this region. They were all employees of the hospital. This study included physical examinations and questionnaires. Functional deficits and degree of disability were analyzed and quantified by “SALSA Scale” (measuring activity limitation) and Safety Awareness Score (indicating an increasing awareness of the risks of certain activities) [1, 2].

## 3. Results

The lesions and their distribution are displayed in Table 1. The vast majority of the lesions were found to be located on the extremities, attributing to the devastating orthopedic effects of this disease. The upper extremity lesions accounted for 32.5%, and the lower extremity lesions were 37.7%. For the anatomical region of the first noted lesion the extremities were the most numerous as well, upper extremity with 33% and the lower extremity with 25%.

Skin sensitivity/neurological testing of lesion via sensation of 291 lesions revealed 64 lesions (22%) to have present/strong sensation, 138 lesions (47.4%) to have present/reduced sensation, and 89 lesions (31%) to not present with any sensitivity. Tactile sense testing revealed 49% of patients were not able to feel wisp cotton over their lesions, whereas 51% were able to. 62% were able to point with one finger directly where the cotton ball had touched, and 38% were not able to. Temperature testing revealed 66% able to recognize both cold and hot temperatures, whereas 34% were not. This displays the severe neurological deficits incurred by the disease process, which lead to orthopedic and surgical complications, as will be explained later in the paper.

The socioeconomic status of the patients was also investigated. Enquiring into years of schooling, 38.9% had 0 years, 40.3% had 1–5 years, 13.9% had 6–10 years, and 7% had more than 10 years of schooling. 52.2% were unemployed, 13% were employed part-time, and 34.8% were employed full-time. Of those that were employed, those that reported their professions can be seen in Table 2. When the patients were asked about their past experience of food shortage, of 45.7% claimed “never,” but 54.3% stated they had “experienced” it at some point in their life. Questions about access to safe drinking water in the last 10 years revealed 70% responded “yes” and 30% responded “no.” Access to sewage disposal in the last 10 years was also investigated, with all responses, 45.7% have access to sewage disposal, and 54.3% never have had that access.

Environmental variables of the patients' lives revealed interesting findings. 100% of the patients lived in a rural setting. From patient responses, 75.7% claimed to live in a home with 0–3 persons per bedroom and 24.3% claimed to live in a home with 4 or + persons per bedroom (of which 4 reported living with 30+ persons per bedroom). 37% of patients have/had animals in the house/yard in the last 10 years, whereas the other 63.1% have not.

Numerous questions were asked about the subject's behavioral patterns as well. Frequency of changing bed linens revealed 14.5% changed linens more than 1 time per 2 weeks, 26.1% with 1 time per 2 weeks, 42% with less than 1 time per 2 weeks, 8.7% with 1 time every 1 month-2 months, 1.4% with 1 time every 3 months, 1.4% changed 1 time yearly, and 5.8% claimed to never have changed their bed linens. 33.8% of patients claimed to share a bed or hammock with others, compared to the remainder who did not. 33.8% of subjects have taken weekly regular baths in open water bodies (creek, river, and/or lake) for the last 10 years, whereas 56.7% have not. Inquires in bathing habits of the subjects reveal that 82% of patients bath greater or equal to one time per day, 11.5% bath greater than 1 time per week, 1.6% of subjects bath 1 time per week, and 5% of subjects never bath on a regular basis.

**Figure 1:**
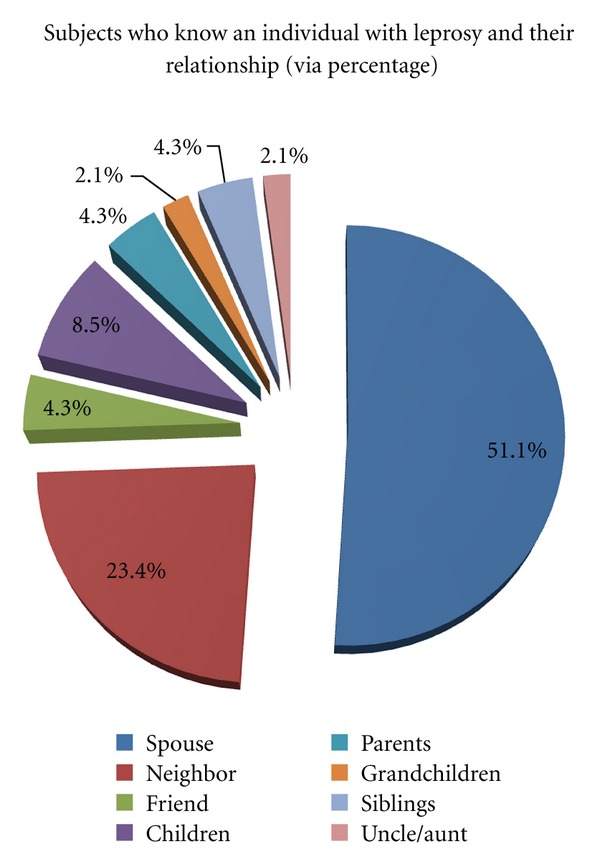


Most of the subjects, 62.7%, claim to know someone else with the disease, with 51.1% claiming that person to be their spouse (see Figure 1). 88.7% proved to be married and 11.3% were not. All of this information allows an individual to draw assumptions as to the living conditions and patterns of those affected and risky behavior which causes increased chance of contracting the disease.

**Figure 2:**
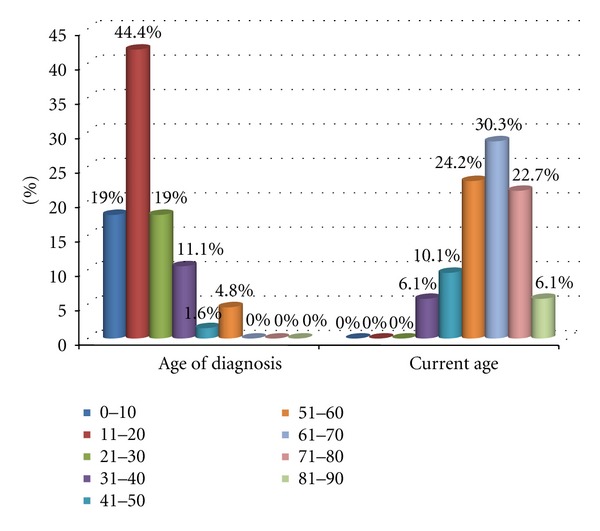


Demographic information of the subjects allows for categorization of our patients into various groups. 57% of the patients were male, and 43% were female. The age of diagnosis is by far greatest for the ages of 0–20. Current age was also collected, and the age group with the largest number of patients, 30.3%, is that of 61–70 years old (refer to Figure 2).

**Figure 3:**
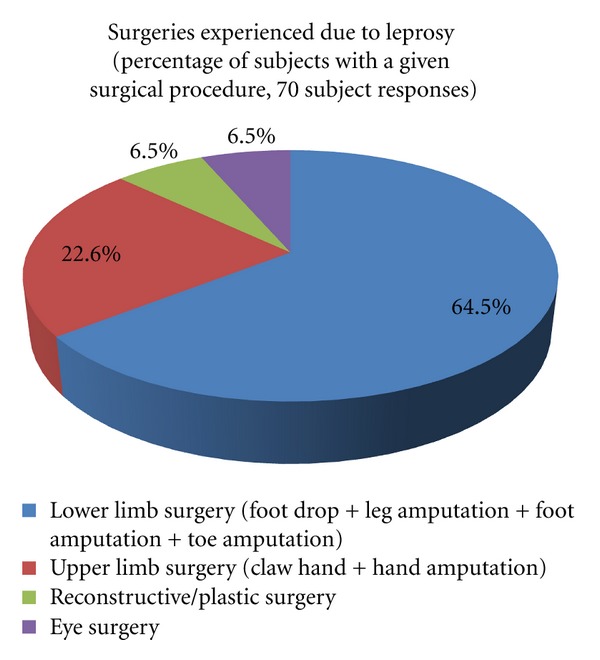


Surgical treatment of the subjects of the study was an area of great interest. 55.70% of the subjects did not received surgery, therefore leaving 44.30% who did receive some form of surgical treatment. Nearly 90% of all surgical procedures were orthopedic in nature, attributing to the severe orthopedic effects of leprosy. Please see Figure 3 for further clarification.

Neurological and disability questionnaire and examination of the patients allowed the researchers to categorize the various lesions into numerous categories. The findings revealed 77% of the patients had a total of 150 separate “disabilities.” Disabilities are defined as anesthesia severe enough to cause functional impairment (18%), ulceration (16.7%), deformity such as claw hand and drop foot (23.3%), absorption/amputation of digit or limb (25.3%), severe loss of vision/blindness (16%), and other (0.7%). Therefore it is clear orthopedic-related disabilities are the most common.

**Figure 4:**
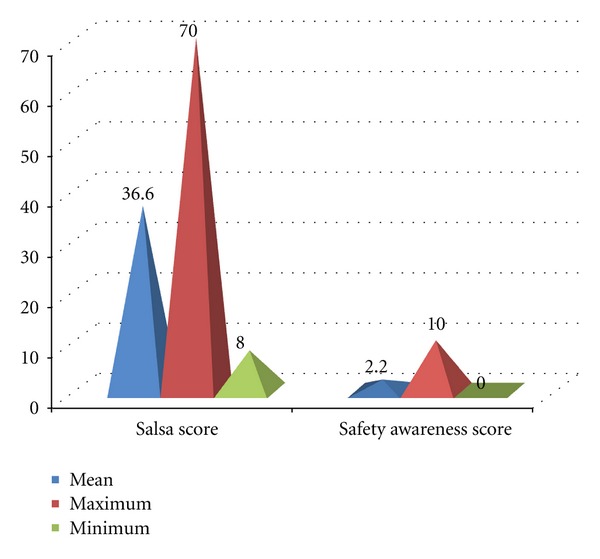


Functional deficits are of great concern when studying leprosy patients; therefore the “SALSA” Scale was used and Safety Awareness Scores were calculated (Figure 4). 

## 4. Discussion

Hansen's disease affects both men and woman in different proportions, but in most regions there is a strong male preponderance of infected individuals. The difference in distribution between the sexes is so drastic that it is very common to have a male-to-female ratio of 2 : 1 in the regions of the world including India, the Philippines, Hawaii, Venezuela, and Cameroon. There is occasionally a larger prevalence among females, as observed in Uganda, Nigeria, Malawi, Gambia, Burkina Faso, Zambia, Thailand, and Japan [1]. This study revealed that 57% of the patients were male, and 43% were female. This is clearly not the 2 : 1 ratio that is known to occur in the country of India. Genotyping of 475 strains of mycobacterium leprae from six different countries has already been completed as most recent studies have shown [3]. There lacks sufficient research as to how many different strains of mycobacterium lepromatosis exist. Each strain of the disease will present with signs and symptoms we collectively call “leprosy,” but each strain has its own unique characteristics in terms of age and sex distribution, and so forth [3]. Due to the fact there is less research in this region of India, the discordance from the 2 : 1 ratio may be due a new undiscovered strain predominating in this region as compared to the rest of India.

Age distribution in previous studies in India and around the globe claims the young are at particularly higher risk for developing the disease than adults [4]. Age of diagnoses of 0–20 years account for well over 50% of all of the transmissions of the disease in our sample population. This study claims the “maximum risk groups” in regards to children are considered to be boys in the age group 5–14 years and females in the age group 5–9 years. This study does not support the theory of a “bimodal age distribution” proposed by other studies, with peak age groups of 10–14 and 35–44 years of age [4–6]. This study displayed a 63.4% distribution between the ages of 0–20 years, which falls in the range claimed by other research to be the age of highest risk of developing the disease. The ages of 21–30 years exhibited a larger percentage of the distribution (19%), than the age groups of 31–90 years combined. This is in contrast to claims of bimodal distribution occurring in the range of 35–44 years as claimed by other studies [5, 6]. These differences in distribution may also be contributed to different strains of the causative agents in this regions compared to others. This further points to the possibility of different strains of the bacteria existing in this region of the country, which has been neglected in terms of leprosy research.

Behavioral and environmental variables allow one to draw certain conclusions as to risk factors in the region contributing to transmission of the disease. Hansen's disease is more often found in rural versus urban settings and is particularly prevalent in areas with tropical climates [7]. Most recent studies show South-East Asia contributes to 69% of the global prevalence and 81% of total new detected cases, India, representing 80% of prevalence and 88% of new detected cases in this region [8].

In order to analyze possible risk factors in terms of transmission, one must compare epidemiological data of this patient population and make correlations with known means of transmission. Transmission is difficult and is thought to occur through the mucosa of the respiratory or alimentary tracts through inhalation or ingestion, via prolonged skin to skin contact (possibly through micro abrasions), through insect bites and via transplacental transmission in rare cases [9, 10]. The investigators found that 24.3% claimed to live in a home with 4 or + persons per bedroom (of which 4 reported living with 30+ persons per bedroom). 33.8% of patients claim to share a bed or hammock with others, and 85.4% of the subjects changed their bed linens less than 1 time per 9 days. Fomites may carry the bacterium for up to 9 days in the tropical climate the study was undertaken in. Most of the subjects (62.7%) claim to know someone else with the disease, with 51.1% claiming that person to be their spouse (see Figure 1). 88.7% proved to be married and 11.3% were not. This information shows a significant proportion of the subjects to be in close physical contact with others. Thus, there is increase probability of coming into contact with nasal secretions, ingesting causative agents, skin-to-skin contact, and fomites with either mycobacterium leprae or lepromatosis. This contributes to transmitting leprosy. It is apparent that the vast majority of the patients knew someone with disease, and most of whom were a spouse. Thus, close contact over a long period of time can be deduced as being a possible risk factor for contracting the illness. Those subjects who had closer relations with a known leprosy patient (e.g., a spouse with the disease versus having a neighbor with leprosy) were at a higher percentage for having contracted the condition. Those who had leprosy and knew someone with the disease who they would naturally come into less contact with (such as an infected neighbor) were smaller in number as compared to those with a spouse with the illness. Thus longer term and closer contact highly increase the risk for transmission. These are all factors which must be taken into account when educating the local population on disease prevention.

Insects are suspected of passing on the bacterium which cause Hansen's disease. 100% of the population lives in a rural environment, which typically contain a larger proportion of insects than that of urban environments. 24 of 65 patients (37%) have/had animals in the house/yard in the last 10 years. This section of the population may lead to increased probability of coming into bacterium carrying fomites carried by or on the animals or bacterium carrying insect on or around the animal. These factors in this region indeed increase the probability of contracting the disease.

Socioeconomic information allows for one to draw further conclusions on the lifestyle of those afflicted with the disease. When this is combined with environmental, behavioral, and demographic information, an individual can paint an overall image of who is typically affected by this disease in the region, and possibly the world. Thus we can draw conclusions as to who is at greatest risk to contract and therefore pass the disease along. Nearly 80% of patients had 0–5 years of formal education. 52.2% of the subjects were unemployed. This information displays a patient population of uneducated and unemployed. Only 34.8% were employed full-time (see Table 2). Some of the professions may even lead to increased risk of transmission and spread of the disease to the surrounding community. These individuals serve as vectors for the spread of disease and are of great concern in disease control. These professions include barbers and tailors, which cause the infected and the clients of these individuals to have close proximity to each other. These therefore increase the probability of transmitting the bacterium via respiratory droplets.

When the patients were asked about their past experience of food shortage, 54.3% stated they had “experienced” it at some point in their life. Questions about access to safe drinking water in the last 10 years revealed 30% claiming “no.” Access to sewage disposal in the last 10 years was also investigated, with 54.3% responding “no.” Leprosy is typically a disease that affects the impoverished and socioeconomically downtrodden, and this paper supports these claims [11].

Dermatological lesions are of much significance in leprosy. They can become the source of anesthesia, open ulcerations, and possible sites of infection. Therefore the importance of recognizing the locations of lesions in leprosy and where they are most likely to occur is of great importance. For the anatomical region of the first noted lesion, the extremities were the most numerous, upper extremity with 33% and lower extremity with 25%. The subject population displayed a total of 240 lesions (36%) were located on the upper extremity, 249 (38%) on the lower extremity, 33 (8%) on the trunk, and 118 (18%) on the face. Abraham's study claims that “distribution of first lesions is nonrandom, confined to exposed parts of the body” [10]. The site of the first lesion is of importance, due to the fact that mycobacterium leprae is thought to be transmitted through skin contact. It has been shown through this study and others that the majority of the first lesions are in locations uncovered by clothing (such as the upper and lower limbs). These areas are vulnerable to abrasion. Abrasions allow for easy entry through the protective layer of skin and into the vasculature of the victim. The bacterium will enter though the abrasion and replicate in macrophages, endothelial cells, and Schwann cells. The replication within and attack of the schwann cells of the local peripheral nerves cause peripheral neuritis [12]. The extremities have a lower body temperature than the rest of the body, which is suitable to support the growth of the organisms [10, 13]. It is only logical the upper and lower extremities would have the largest number of lesions and thus the largest number of healing lesions as well.

Through the various means of transmission, the causative agents of leprosy can enter into the body of the victim. The bacteria are obligate intracellular parasites, with a tropism toward inhabiting and replicating within macrophages, endothelial cells, and Schwann cells [14]. It is the high degree of affinity the bacterium has toward the schwann cells of the peripheral nervous system which sets the stage for much pathology to occur [15]. Schwann cells have numerous roles in the peripheral nervous system. A fraction of these roles include the ensheathment and myelinate of axons to ensure the ability of nerve impulses to travel along axons, regulation neuromuscular junction synaptic activity, and regeneration nerve fibers [16–18]. Therefore the infiltration and effects the bacterium have on the schwann cells of the peripheral nervous system affect these roles. As a result, nerve conduction of cutaneous fibers relaying information regarding skin sensitivity, tactile sensation, and temperature awareness are all altered in the disease of leprosy. The body becomes anesthetic due to the destruction of sensory fibers contained within the peripheral nerves. This results in the affected areas becoming prone to the injuries that result in open wounds and ulcerations. Leprosy victims are not aware of self-incurred injuries, leading to neglect of surface injuries, leading to progressive ulcers and sites of infection which may spread, leading to osteomyelitis in cases and need for amputation [15].

78.4% of the lesions had some degree of altered sensation. The degree of impairment to sensation is thought to largely correlate to the degree of infiltration of the bacterium into Schwann cells. Peripheral sensory fibers include those of cutaneous touch as well as temperature. Thus the damaging effects on these nerves would display effects on both sensations due to the neurological effects of leprosy [19]. These neurological effects are explained in the Section 3 of the paper. The findings of this study display differences in the neurological damage as compared to similar studies. Past studies have revealed as much as 93% of the participants displayed touch sensory and 44% thermal sensory impairment [20]. This difference also leads credence to the proposition of a different strain of the bacterium as the causative agent of the disease.

The limbs become anesthetic, ulcerated, possibly infected, paralyzed, and deformed [15]. Deformities such as claw hand and drop foot are common in this disease, and amputations are not uncommon. Pyogenic organisms readily enter through the lesions in the skin. This may lead to abscesses and resultant treatment of bone, joint, and tendon sheath infections, along with enucleating the eye when its globe has become infected. Ophthalmological complications lead to numerous surgical treatment measures [15]. Neuritis and ulcerations of the skin lead to numerous lesions which indicate reconstructive and plastic surgery on many leprosy patients, most commonly saddle-nose deformity nasal reconstruction and skin grafts.

Of the patients population, 44.30% of population claimed to have some form of surgery in the past due to the effects of the disease. Orthopedic surgical procedures on the extremities proved to be the most numerous, comprising 87.1% of all of the surgeries performed. This can be contributed to affinity of the disease causing bacterium toward the cooler temperatures of the peripheral nerves. This results in many of the manifestations of the disease which subsequently appear in the extremities [21]. This information is vital in identifying and therefore formulating measures to avoid extremity injury and surgery.

77% of the study population reported some form of disability. 48.6% of the subjects indicated some form of disability in the extremities, as a deformity (e.g., claw hand and drop foot) or absorption/amputation of a digit or limb. We can plainly see majority of injury caused by the disease is orthopedic in nature, which is ignored by many previous studies. One may conclude from the data stated above that the majority of the disabilities incurred due the disease were to the extremities, and the majority of those disabilities are regarded as the most severe in nature according to the grading criteria. When comparing data displaying the location of first lesion, the average number of lesions per anatomical region, and the number of lesions in the healing stage, all findings indicate the extremities to display the most numerous findings. It is no wonder why the vast majority of surgeries performed on the patient population were orthopedic in nature (87%).

Functional deficits are of great concern when studying leprosy patients, the degree of which is of direct relation to many of the orthopedic sequela of the disease. The Screening of Activity Limitation and Safety Awareness (SALSA) questionnaire is a 20-question assessment measuring patients understanding for the problems they encounter amongst the activities of their daily life. The scale itself is the product of collaborative research in five countries in four continents. It is used throughout the world as a means to rank the functional deficits as incurred by the disease of leprosy. In proper attainment of a SALSA score, a 20-item SALSA questionnaire allows a score of 0 to 4; 0 = “I do not do this activity,” 1 = “the activity is easy to carry out,” 2 = “the activity is a little difficult to carry out,” 3 = “the activity is very difficult to carry out,” 4 = “I physically cannot do the activity” or “I avoid the activity because of risk” [22]. From the 20 questions a score for SALSA and safety awareness can be collected. If a score of 20 is obtained one can assume the patient performs all tasks easily while the maximum score of 80 indicates the extreme hardships the patients must go through when performing the activities of daily life. When obtaining the safety awareness score, a value between 0 and 11 is possible. An elevated score displays the patients understanding for the level of risk they are exposed to with each activity they participate in and also indicates the limitations they place on themselves due to these increasing risks [23]. Of the SALSA scores calculated, the mean was 36.6, the maximum value attained from an individual was 70, and the minimum was 8. In a recent study in Africa, with over 100 patients used in the sampling process, average values of 36.5 and 35.02 were attained. In another study in Brazil, the majority of the patients displayed a score hovering around 37 [24]. These values are in very close relation to the data collected by this study. This may indicate that although there are different pathological mechanisms of the different strains of the disease causing bacterium, there are similar results in the affects of functional impairment caused by leprosy. This midlevel mean score of 36.6 on the SALSA scale may display a moderate level of functional affects. This translates to a mean value of 2.2 (of a possible maximum value of 11) for the safety awareness score. Although the mean was 2.2, amazing 50% of subjects displayed the minimum score of 0. This indicates a low understanding for the level of risk they are exposed to with each activity they participate in and also indicates the limitations they place on themselves due to these increasing risks, as stated before. This could be due to the poor education of the patient population by their medical providers, or a disregard of the information provided to the patients. In the poverty stricken country of India, this disregard may be due to the need to provide a means of living to the patients and therefore a need to stay physically active.

## 5. Conclusion

The demographic factors like age and sex distribution of the leprosy patients in this region of the world are in conflict with previously stated rates and figures. The degree of neurological deficits is also different from those found in previous studies. The authors of this paper claim a new undiscovered strain of bacterium to be the causative agent of these differences. It is a well known fact that different parts of the world have different causative agents leading to the varying clinical presentation known as leprosy. This is a very unique and substantial proposal. Clinical data points to the possibility of a new strain of the disease. The authors call on future research to identify this unique strain. This information is of significance because it effects prevention and improved treatment of the disease, which leads to devastating orthopedic implications as quantified and displayed in the paper by lesions, functional and neurological sequela, disability ratings, and the need for surgical intervention.

This study has clearly displayed the epidemiological data of this patient population. This data has identified risk factors contributing to the spread of this horrendous disease. The vast majority of the infected individuals of this particular region could be significantly protected if educated of the risk of contracting the disease from those they are most often in contact with. For example, health precautions should be taken for many of the infected individuals working in professions which may lead to higher incidences of transmission of the disease.

Despite the treatment, the disease and surgical procedures have left in their wake, a functionally impaired population as displayed by the numerous surveys in this study. This calls upon local health officials to provide increased treatment in terms of prosthesis and rehabilitation to this patient population. It also shows that a large percentage of the patient population has been profoundly affected by the disease, in that it had progressed to a degree in that surgery was needed as treatment. Thus, strict control should be maintained over the patient population and public education about the disease to lessen the transmission rates and decrease the maleffects of the illness.

Many of the proposed pathological mechanisms of the disease and vast majority of the pathological sequela have proven to be orthopedic in nature. This fact is not highlighted in the vast majority of medical literature. This study revealed many of the epidemiological risk factors associated with contracting the disease, the pathology, the sequela of the disease as they pertain to orthopedics and surgery most notably, preventative measures, and treatment options. As stated before, all the data can be used to form prevention and treatment strategies in order to prevent the orthopedic and surgical-related sequela of leprosy abroad, but especially in this region of the world which lacks previous research.

## Figures and Tables

**Table 1:** 

	Average number of lesions per anatomical region (113 responses)	Number of subjects w/lesions in that particular anatomical region (113 responses)	Number lesions in healing stage, per anatomical region (77 lesion in total recorded in the healing stage)	Percentage of lesions in healing stage, per anatomical region (77 lesion in total recorded in the healing stage)	Anatomical location of first lesion
Scalp	1	11	0	0%	0%
Face	1	3	3	4%	15.60%
Neck	1	5	0	0%	0%
Chest	2.4	5	3	4%	3.10%
Back	1.9	10	9	12%	9.40%
Pelvis	1	2	2	2.6%	1.60%
Buttock	1.3	8	6	7.8%	6.30%
Upper arms	2.23	13	7	9.1%	20.30%
Lower arms	1.6	13	10	13%	4.70%
Hands	2.5	12	8	10.4%	7.80%
Thighs	2.4	5	5	6.5%	2.50%
Legs	2.2	12	9	12%	9.40%
Feet	2.1	14	15	19.5%	4.70%
Upper extremity	2.1	38	25	32.5%	32.8%
Lower extremity	2.2	67	29	37.7%	16.6%

**Table 2:** Reported Professions.

	Percentage of subjects w/that particular profession
Agriculture (ex. farm hand)	36.40%
Food stall	6.10%
House keeper	6.10%
Tailor	9.10%
Textile worker	3%
Railway bag clerk	3%
Cook	3%
Shop keeper	3%
Cigarette factory employee	3%
Business man/woman	3%
Construction	3%
Hotel clerk	3%
Clothing store employee	3%
Grocery store employee	6.10%
Security guard	3%
Barber	3%
Gardener	3%
